# First wide-angle view of channelized turbidity currents links migrating cyclic steps to flow characteristics

**DOI:** 10.1038/ncomms11896

**Published:** 2016-06-10

**Authors:** John E. Hughes Clarke

**Affiliations:** 1 Department Geodesy and Geomatics Engineering, University of New Brunswick, P.O. Box 4400, Fredericton, New Brunswick E3B 5A3, Canada

## Abstract

Field observations of turbidity currents remain scarce, and thus there is continued debate about their internal structure and how they modify underlying bedforms. Here, I present the results of a new imaging method that examines multiple surge-like turbidity currents within a delta front channel, as they pass over crescent-shaped bedforms. Seven discrete flows over a 2-h period vary in speed from 0.5 to 3.0 ms^−1^. Only flows that exhibit a distinct acoustically attenuating layer at the base, appear to cause bedform migration. That layer thickens abruptly downstream of the bottom of the lee slope of the bedform, and the upper surface of the layer fluctuates rapidly at that point. The basal layer is inferred to reflect a strong near-bed gradient in density and the thickening is interpreted as a hydraulic jump. These results represent field-scale flow observations in support of a cyclic step origin of crescent-shaped bedforms.

## Abstract (web summary)

The basal structure of turbidity currents and their association with crescent-shaped bedforms has not been observed at the field scale. Here, the author presents views of turbidity currents moving over and modifying such bedforms in a manner consistent with theoretical and laboratory studies of cyclic steps.

## Graphical abstract

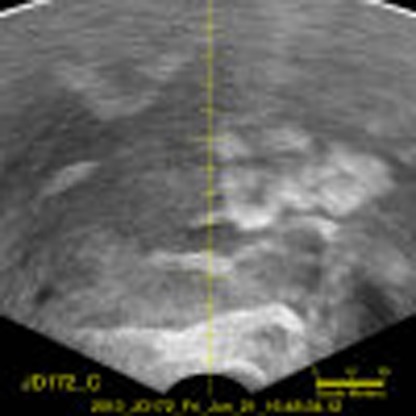

Submarine sediment-laden flows called turbidity currents transport the majority of terrestrially derived sediments that fill the world's ocean basins^1^ and can build enormous channel networks on the deep ocean floor^2^. As such, they are volumetrically and morphologically as significant as the sediment flux of all the rivers on earth. While the manner in which sediment is moved in rivers is well understood with hundreds of thousands of field observations, to date there are remarkably few observations of marine turbidity currents^1^. Considerable uncertainty therefore remains over what turbidity currents are, particularly their density structure, and how they build and modify the constraining channels and underlying seabed relief.

Submarine fibre optic cables that carry over 95% of the world's internet traffic lie across the path of turbidity currents and are often interrupted by their passage^3^. As oil and gas reservoirs beneath the continental shelf and slope are being increasingly depleted, the search for hydrocarbons has moved into deeper waters, where modern turbidity current flows present a potential hazard to oilfield infrastructure. Better knowledge of the characteristics of these flows could thus help mitigate the hazard to deep-sea infrastructure.

The three main reasons why there are so few observations of marine turbidity currents are their sometimes infrequent and irregular reoccurrence interval (10^2^ years or more), relatively inaccessible depths and often destructive nature. Fjord delta environments, however, have been shown to be excellent locations^4^ for monitoring turbidity currents, in which the recurrence interval can be much more frequent and in shallower depths that are easier to instrument.

Numerical modelling^5^ and flume experiments^6^ have clearly shown that the primary controlling factors for turbidity currents are the density and velocity profiles, especially close to the base of the flows, where the highest gradients in both exist. In the absence of detailed flow information beyond laboratory-scale experiments and slower continuous sediment-laden underflows in lakes^7^, most previous inferences of the mechanisms of turbidity current initiation, flow and deceleration were based predominantly on observations of the resulting deposit. To explain the deposits, end member mechanisms involving high- or low-density turbidity currents have been invoked^8^,^9^. For more powerful seafloor-altering flows, a high-density basal layer is often inferred, although evidence for such a feature has only ever been seen in the laboratory^10^,^11^.

In fluvial environments, good correlation between observed bedforms and measured stream power has been demonstrated^12^. For the majority of deep-sea channels and lobes, the bedforms are often the only preserved morphologic evidence and thus have been used to infer the nature of the flow^13^. However, unlike open channel flow (fluvial) bedforms, the migration of bedforms under submarine density flows has only previously been observed at laboratory scale^14^. Thus, inferring flow characteristics using just bedform morphology and analogies with open channel flow^15^,^16^ can be ambiguous, as super and subcritical flow can sometimes generate similar resulting relief.

Of particular current interest are so-called crescent-shaped bedforms (CSBs) observed in many deep-sea channel systems worldwide. Their origin has been attributed to a variety of mechanisms, including internal tide currents^17^, rotational slumping^18^, antidunes^19^, as well as Froude supercritical cyclic steps^20^. The cyclic step hypothesis^21^,^22^ is being increasingly favoured based on morphological evidence^23^. Before the results presented here, however, the flow associated with actively migrating CSBs had never been observed.

Three-dimensional seismic imaging of the subsurface structure of CSBs^24^ suggests that are upslope-migrating features. With the advent of repetitive multibeam surveys of active turbidity current channels, similar CSBs from several localities have been clearly demonstrated to migrate upslope with time^25^,^26^,^27^, implying supercritical flow. Previous work at this location using an unusual set of 93 daily repeat surveys^28^ has shown unambiguous upslope migration of trains of CSBs within the channels (Supplementary Movie 1).

Flume experiments examining sedimentation over cyclic steps under the body of subaqueous sediment-laden density flows^14^ have illustrated that a denser basal layer should be present. Furthermore, the flow thickness and speed of that layer should be strongly modulated with a hydraulic jump (HJ) present just downstream of the base of the lee slope.

Herein, the most detailed *in situ* view to date of active turbidity currents in any environment is presented. Using a novel acoustic-imaging geometry, the instantaneous appearance of the head, body and wake of active flows are observed for the first time in both plan view and along channel sections. This allows the spatial and temporal evolution of flows to be examined in unprecedented detail. The flows are monitored as they move within a submarine channel over CSBs that are clearly altered by the passage of the flow. The imagery reveals the flow modulation over a complete bedform and shows a distinct basal layer, during the period when the bedforms are actively migrating that thickens abruptly just after the base of the lee slope. These observations support the hypothesis that cyclic HJs in the flow are responsible for the development of these CSB's.

## Results

### Experimental configuration and timing

**Figure 1:**
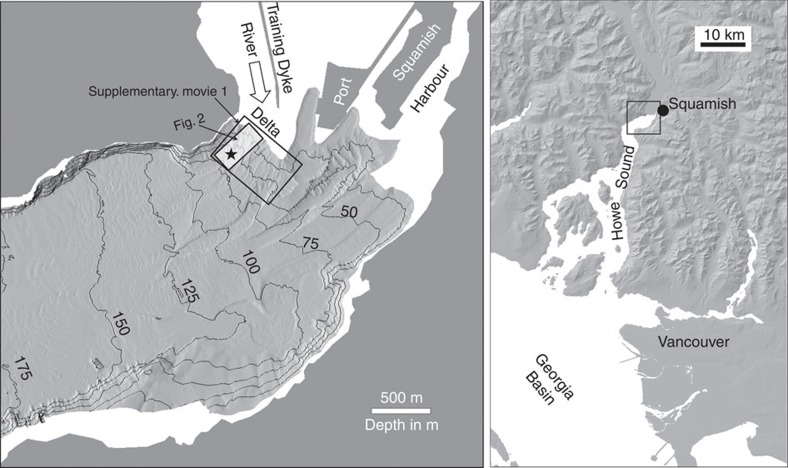
Location map. Location map showing the relationship of the Squamish River to Howe Sound and the bathymetry seaward of the delta. Inset rotated box shows the location of Fig. 2. Star indicates instrument location. Larger rotated box indicates location of Supplementary Movie 1.

The experiment took place on the prodelta slope off the mouth of the Squamish River (Fig. 1) that drains into Howe Sound, British Columbia, Canada. The Squamish River discharges >1 million cubic metres of sediment per year^29^ and that flux has been artificially constricted since 1971 by a training dyke to allow for the development of the adjacent port. As a result, on the prodelta in the vicinity of the experimental site, an average sedimentation rate of ∼1 m per year is typical^30^.

**Figure 2:**
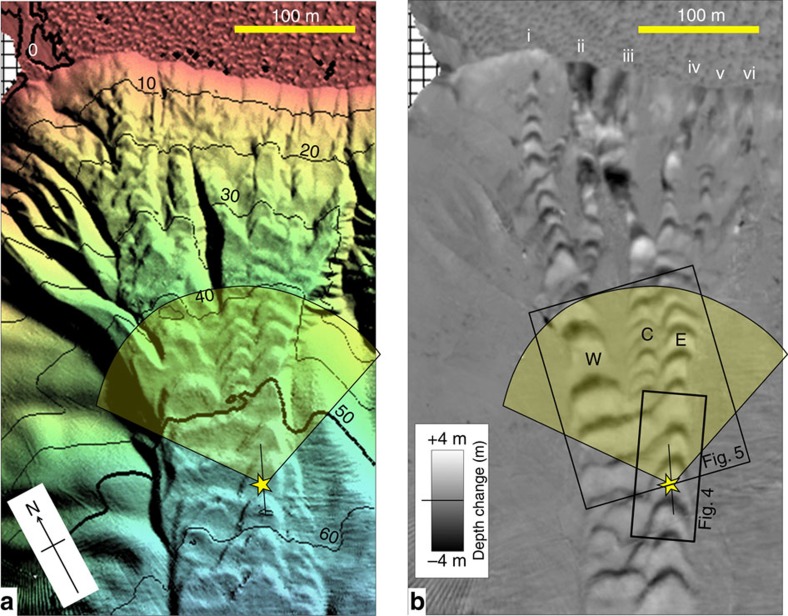
Bathymetry and seabed change. Bathymetry and seabed change from lip of delta to 70-m depth along the North Channel of the Squamish prodelta (rotated box in Fig. 1). (**a**) Bathymetry after the lower low water at end of 21st June, 10-m contour intervals, referenced to chart datum. (**b**) Bathymetric change between end of 21st June and end of 20th June. Star indicates the location of the instrument package. Superimposed is the location of the 150 m radius 120° sector imaged in plan view, as well as the 34-m long transect imaged in section. Roman numerals refer to starting point of mass-wasting trains.

The northern channel, in which the experiment took place, is fed from a convergent series of gullies, extending down from a concave section of the delta lip (Fig. 2a). The floor of the channel is covered with CSBs arranged along multiple parallel thalwegs. The instrument package used in this experiment was installed suspended above the eastern thalweg of the channel (Supplementary Fig. 1). At that point, the channel lies at a depth of 60 m. It is ∼85-m wide and the average relief of the constraining channel flanks is ∼4 m. The channel floor relief is strongly modulated by the bedforms with a local wavelength of ∼30 m and height of 2–3 m. The mean slope of the channel floor at the point of measurement is ∼5°. At that point, the bedforms locally modulate the slope from ∼25° on the lee slopes to −3° on the stoss sides. The instrument package was located 300 m from the lip of the delta that is 1 m below low water (chart datum). The regional channel floor slopes increase up to the lip, being ∼10° at a distance of 100 m from the delta edge, and the maximum slope of the delta edge is about 35°.

**Figure 3:**
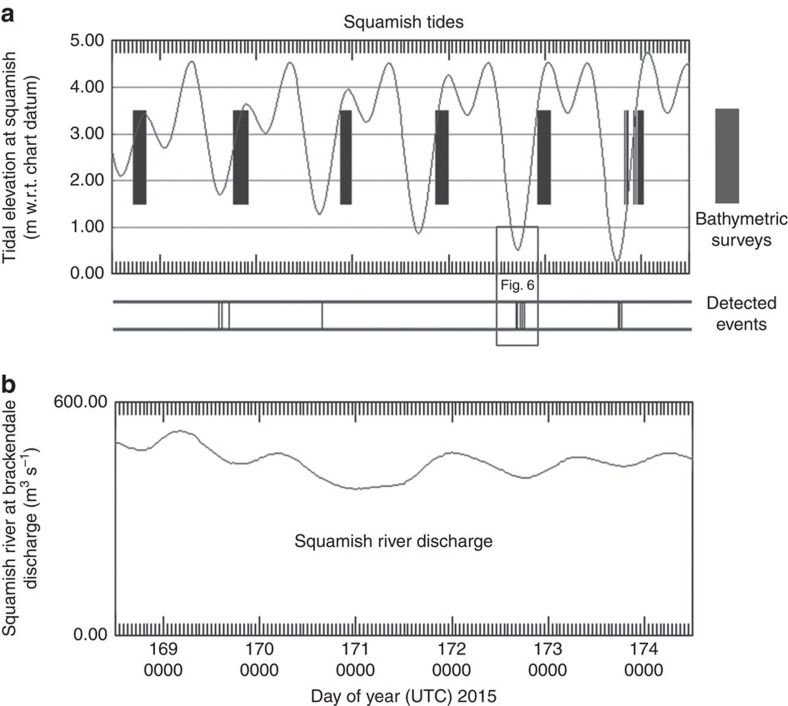
Event timing relative to river discharge and tides. Time series of tidal height river discharge (lower) and river discharge (upper) during the 6-day experimental period. Shaded regions represent acquisition window of the bathymetric surveys. Central bars indicate timing of detected turbidity current events. Inset box indicates time period of Fig. 6.

Over a period of 6 days in June 2013, the instrument package was suspended above the channel. During that period, the river discharge maintained a typical summer level of ∼450–550 m^3^ s^−1^ (Fig. 3). The area has a mixed semi-diurnal tide and previous field programs^28^,^31^ had indicated that the turbidity currents were most common within an hour of lower low water (LLW) when the off-delta river currents peak. The experiment was specifically designed to cover the period as the tides evolved from neap to spring conditions (Fig. 3) and thus the LLW level decreased daily and the corresponding off-delta flow increased. A suspended acoustic Doppler current profiler (ADCP), deployed 24 h per day, confirmed that no events happened >1 h away from the LLW. During the 3-h window around LLW, the suspended imaging multibeams and a lowered probe were additionally deployed to examine the events in more detail.

Over the 6 days, 14 turbidity current events were detected and all occurred within 1 h of the LLW (Fig. 3). On 21st June (JD172), the greatest number of events (7) were detected and the most significant seabed change was measured (Fig. 2b). The present paper focuses on the fastest four of those seven events, comparing and contrasting their relative speed, plan form, duration, thickness, suspended sediment content and associated seabed change.

### Bathymetric change detection

A single multibeam bathymetric surface was obtained each day after the LLW on the rising and high water period (Fig. 3). These surfaces were used to quantify the net bathymetric change associated with all the turbidity currents that were triggered in the preceding 24 h period. As no flows were ever detected outside the LLW window, these changes could be entirely attributed to the cumulative result of the observed turbidity currents on that day.

**Figure 4:**
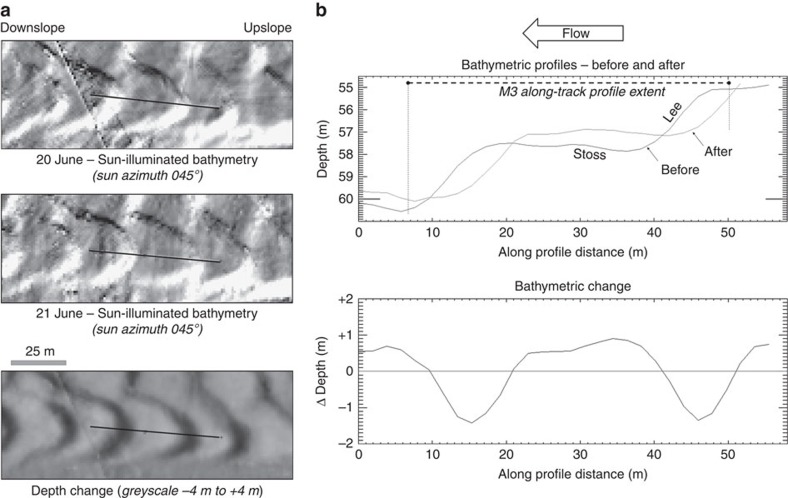
Detail bedform relief. (**a**) Sun-illuminated topography before and after the low water period, and difference map between the two surfaces. Location indicated in Fig. 2. (**b**) close up of detailed bathymetric profiles and relative change directly underneath the suspended instrument during the 21st June low water period.

The net bathymetric change associated with the 21^st^ June LLW period, when the seven flows were detected, is presented in Fig. 2. As can be seen, the CSBs on the floor all migrated upslope. At the location of the instrument, there are two main thalwegs that form from the coalescence of three thalwegs (W,C and E in Fig. 2) that exist at the furthest upslope view of the suspended multibeam. The bathymetric difference map (Fig. 2b) indicates that those three channels were fed from six main locations just below the delta lip (Fig. 2b, i–vi). The bathymetric change directly under the suspended instruments is shown in Fig. 4, with a profile extracted along the transect corresponding to that viewed by the downward-looking multibeam. The change in the bathymetric profile before and after the low water, clearly indicate a 5–7-m upslope migration of the bedform with 0.4–0.8 m of deposition on the stoss side and a peak of 1.6 m of erosion on the lee face.

### Planiform multibeam imaging

**Figure 5:**
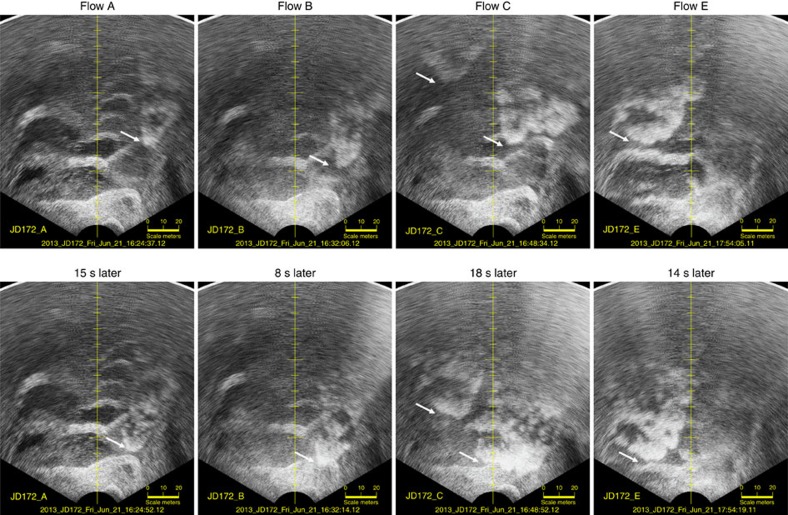
Plan view of approaching turbidity current heads. Two sequential plan views ∼10–15 s apart, of the four most active turbidity currents on 21st June, as seen from the forward-looking imaging multibeam. Maximum slant range is 150 m. White arrows indicate the leading edge of the flow. Location of area is indicated in Fig. 2.

The forward-looking imaging multibeam could view all the channel thalwegs and bedforms in plan view to a range of 150-m up-channel. The approaching head of the flow could be clearly monitored propagating towards the multibeam (Fig. 5; Supplementary Movie 2). As the plan view included the full width of the channel, it is possible to assess whether the flow originates from one or more of the three thalwegs in view.

The leading edge of the flow was the most distinct with a characteristic protruding nose that follows the middle of the thalwegs. In the wake of the flows, the bedforms were obscured for several minutes suggesting that the remnant suspended sediment is blocking the acoustic propagation.

By tracking the leading edge of the flow, the head speed could be directly measured. The four flows presented (A, B, C and E) all moved faster than 2 ms^−1^. Flow A, in the eastern thalweg, was the slowest with an average speed of 1.9 ms^−1^ and was decelerating from 2.4 to 1.4 ms^−1^ over the 150 m section. Flow B was also in the eastern thalweg and averaged 2.6 ms^−1^, while at times surging to over 3ms^−1^. Flow C consisted of three converging flows in differing thalwegs. The heads of the flows in the central and eastern thalweg converged and passed under the instrument package almost simultaneously and the flow in the western thalweg was ∼15 s behind. Over the 150 m section, all three flows averaged ∼2.5 ms^−1^ while surging up to 3.0 ms^−1^. Flow E consisted of two flows with a slower preceding flow of ∼1.6 ms^−1^ in the eastern thalweg, followed by a wider and faster flow of ∼2.5 ms^−1^ in the western thalweg that arrived ∼1 min later.

### Vertical profile imaging

The downward-looking multibeam examines an along channel cross-section immediately below the instrument and thus would only see flows that occurred in the eastern thalweg. Thus for example flow D, while a significant flow as seen in planiform, passed by solely in the western thalweg and therefore only a minor signature was observed from the downward-looking multibeam. Similarly, the downward-looking view of flow E best records the passage of the preceding weaker flow in the eastern thalweg, and provides only a partial view of the stronger flow in the western thalweg 60 s later.

**Figure 6:**
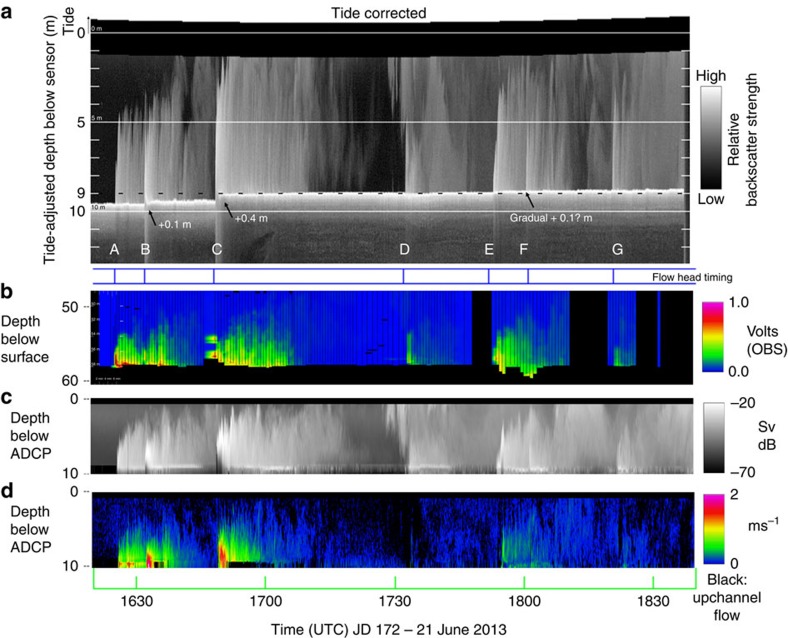
Time sequence of all seven events. Showing the timing of the seven discrete turbidity currents events (A–G) during the 2 h 20 min period around low water on 21st June. (**a**) Downward-looking 500 kHz M3 nadir intensity profile. The profile is plotted, corrected for the change in instrument depth due to the tide. The brightest echo is the seabed, showing the abrupt net accretion within the∼2 min period of event C. The nine metre contour (dashed line) is highlighted to aid in detecting erosion or accretion. (**b**) Relative optical backscatter profiles (volts) for the same period. (**c**) ADCP 1,200 kHz acoustic volume scattering strength (Sv)^41^ and (**d**) along channel component of the flow velocity as defined by the ADCP.

**Figure 7:**
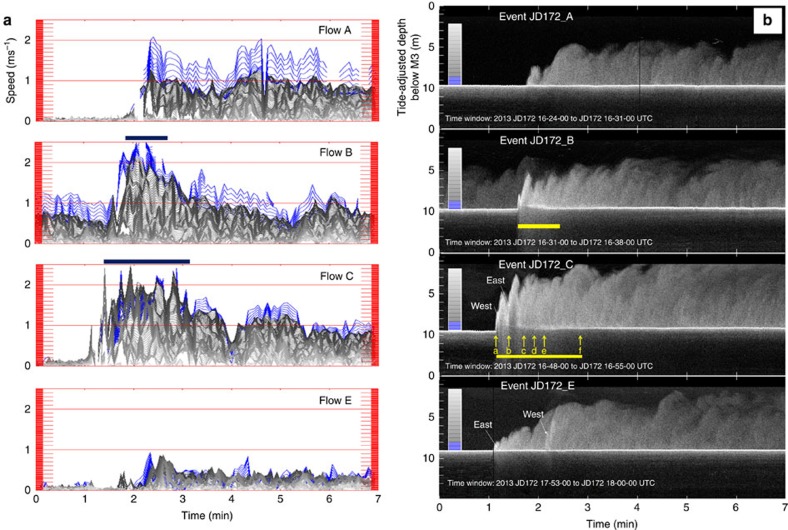
Velocity structure and acoustic scattering sections. Expanded 7-min time series for the strongest four flows (A, B, C and E) of the seven events during the 21st June low water period. (**a**) ADCP velocities from 8.5 m above the seabed to within 0.5 m of the seabed. The line darkness increases from the top of the flow downward. The bottom 1 m of the flow is indicated by blue lines. Although the data were recorded at 25-cm intervals, the profiles are interpolated at 5-cm intervals to better illustrate the gradients in the flow structure. Solid black bars indicate period of Supplementary Movies. Note that flow B is running into the back of the wake of flow A and thus the velocities before the head are nonzero.(**b**) 500 kHz acoustic scattering intensity profiles derived from the centre beam of the M3 downward-looking multibeam. Yellow arrows in flow C represent times of the still vertical sections presented in Fig. 8. Solid bars represent the 50 and 125 s period of the supplementary downward-looking movies provided. West and east indicate the flow surges from the west and eastern thalwegs respectively.

The downward-looking beam of the vertically mounted multibeam was used to create a centimetre resolution vertical profile time series of 500-kHz acoustic volume scattering strength. A 2.3-h long record of that profile is presented (Fig. 6a), showing the passage of the seven events on June 21st. Figure 7b shows an expanded 7-min time series for the four most significant events. As can be seen, the flow thicknesses, based on the maximum height of suspended scatterers, ranged from 3 to 7 m. For all flows apart from the fastest two (B and C), the sediment–water interface under the flow remained clearly visible at all times. Uniquely for flows B and C, the suspended sediment concentration in the flow rose to a level sufficient to attenuate the acoustic energy returning from the sediment–water interface below. As a result, below the top of the attenuating layer, the scattering signal fades away with depth and the corresponding diffuse peak in the intensity records a level in the flow at which the sediment concentration goes over a threshold, rather than the sediment–water interface.

Previous laboratory studies^32^,^33^ indicate that the suspended sediment load would need to be at least of the order of 50 kgm^−3^ before acoustic attenuation becomes sufficient to prevent further acoustic penetration. The exact level, however, would be a function of frequency, grain size distribution and cumulative attenuation in the layers above. Thus, while this horizon can be considered a density layer, it cannot have a precise suspended sediment concentration associated.

The height above the seabed at which that attenuation occurred varied through the duration of flows B and C, indicating that the instantaneous location of that density layer in the flow was undulating. As the flow waned, the seabed below reappeared, indicating that the required concentration of suspended sediment was no longer present.

The instrument package was suspended at a constant height from the sea surface directly above the nearly flat part of the stoss side of the bedform (Fig. 4), and the reference point on the vessel, from which the package was suspended, did not move laterally >1 m during the whole 2.3-h period. Thus, with tidal correction, the change in the seabed elevation directly below the sonar can be precisely monitored. The 2.3-h time series of the vertical profile (Fig. 6) clearly indicates that the seabed elevation was almost unchanged through the period with the notable exception of flow C. During the 2-min window when flow C exceeded 2 ms^−1^, the seabed accreted ∼0.4 m closely corresponding to the net bathymetric change seen over the full low water period (Fig. 4). Smaller shifts of∼0.1 m (close to the limit of resolution) are seen at the start of flow B and a more gradual accretion through the wake of flow E (Fig. 6a).

### Along track profile evolution

**Figure 8:**
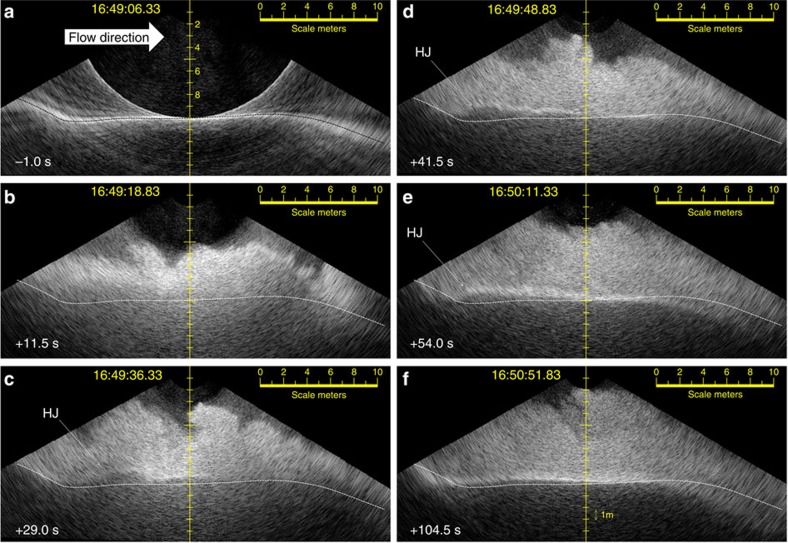
Vertical sections along length of bedform. For flow C, six still frame M3 imaging sections along channel over the length of a single bedform. Flow from left to right. (**a**) Frame immediately before first appearance of flow head. (**b**) 11 s after flow head arrival showing attenuated signal, indicating highest sediment concentrations above the bed. (**c**,**d**) Successive frames showing first and subsequent clear view of hydraulic jump (HJ) developed on top of basal attenuating layer. (**e**) Waning view of hydraulic jump that has now propagated towards the base of the lee slope. (**f**) Waning low-density flow after which point no seabed alteration is observed. The location of the seafloor immediately before onset of flow is indicated by a black dashed line in (**a**). For subsequent frames, that profile is highlighted as a white dashed line to appreciate the frame to frame displacements. Note the net upslope shift in the bedform lee slope location from (**a**–**f**).

The downward-looking M3 multibeam recorded the along channel topographic profile and acoustic volume scattering from the water above over a ±60° sector. This corresponds to a section ∼34 m long covering the length of a single bedform centred over the stoss side and extending from the preceding upstream lee face to the downstream lee slope (Fig. 8). Over the 2.3-h period of observation, while the instrument oscillated in heading∼±2°, the mean heading drifted only 1.5° over that duration. Thus, the underlying section can be assumed to be effectively stationary. Because the internal pitch sensor of the instrument had an overprinting noise signature, the fore-aft rocking of the profile had to be stabilized using image-to-image correlation. Despite that, the time series of the along track profile can be considered a stable reference to examine the evolution and migration of the single bedform through each flow passage.

For flow C uniquely, the lee face was clearly seen to migrate upslope (compare Fig. 8a,f). For all other flows, the bedform profile immediately before, and subsequent to, each flow event was not resolvably different.

In addition to the shift in the sediment–water interface, for flows B and C that exhibited an attenuating layer, the elevation and shape of that layer varied across the bedform (Supplementary Movies 3 and 4). Representative stills of flow C in Fig. 8 (and flow B in Supplementary Fig. 2) illustrate the variation in the thickness of the attenuating layer along the length of the bedform. For flow C, the elevation of the attenuating layer can be seen to fluctuate with two pulses (Fig. 7b flow C, east and west), corresponding to the passage of the two flows in the east and west subchannels ∼15 s apart (Fig. 5 flow C; Supplementary Movie 2).

For about the first 20 s of flow C, the attenuating level was 2–3 m above the seabed and had an oscillating upper surface (Fig. 8b). Subsequently, for about another 60 s, a much more planar-attenuating layer was observed on the stoss side that thinned down current from 1.0 to 0.5 m above the initial seabed (Fig. 8c–e). That interface fluctuated rapidly only downstream of the lee face of the bedform. At that point, the interface abruptly thickened downstream (HJ in Fig. 8). The location and shape of that thickening was not stable, altering over a time scale of a few seconds (Supplementary Movies 3 and 4).

For flow C, some of the lee face migration occurred within the first 20 s when the elevated attenuating layer was obscuring the flow. For the following 90 s, the lee face migration continued accompanied by net accretion on the stoss side downstream of the layer thickening. As the flow decelerated, the point of thickening (indicated by HJ in Fig. 8) migrated progressively closer to the base of the lee face, while reducing in amplitude.

### ADCP flow velocities

At a horizontal offset of 15 m down flow from the M3 multibeams, a 1,200 kHz ADCP was also suspended. Through examination of the difference in depth recorded in the four individual beams (Supplementary Fig. 3), it is clear that the ADCP was centred over the lee face of the next bedform downslope. As a result, valid velocities can only be obtained above the minimum vertical distance to the seafloor (Methods). That closest point on the seabed was 10 m below the instrument. Velocities reported below that elevation should be treated with suspicion (Methods).

**Figure 9:**
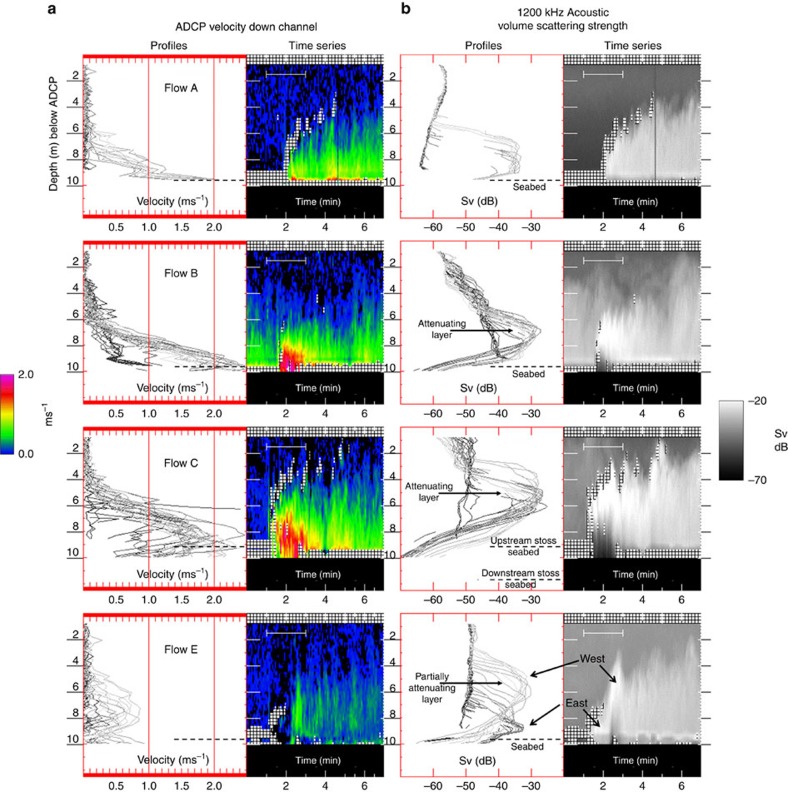
ADCP velocity and acoustic scattering. Time series plots of the 1,200 kHz ADCP down channel velocity (**a**) and acoustic volume scattering strength^41^ (**b**) for a 7-min time window during the strongest four flows (A, B, C and E). Vertical profiles for the peak 2-min period (indicated by horizontal bar in time series plot) are extracted every 8 s using 0.25 m layers. Plotted line grey level represents sequential profiles from black (first) to light grey (2 min later). Seven-minute time window is identical to that in Fig. 7.

For the same 7-min period, time series of ADCP current velocity are shown for heights up to 8.5 m off the minimum seabed distance (Fig. 7a). For flows A, B and C, the largest velocities were most commonly restricted to the bottom 1 m above that seabed (coloured blue in Fig. 7a). Notably, though during the period when the attenuating layer is present in flows B and C, data within 1 m of the seabed, are either absent or anomalously slow. The 2.5–3.0 ms^−1^ velocities derived from the head propagation for flows B and C are only intermittently recorded by the ADCP (Fig. 9a), suggesting that those velocities were primarily achieved in flow below the attenuating layer.

The characteristically asymptotic drop in speed with elevation in the upper interface can be clearly seen (Fig. 9a). Due to sidelobe contamination that extends up to 0.50 m off the bed, and the attenuation of sound at higher sediment concentrations (Fig. 9b), the velocity structure of the basal boundary layer could not be examined. Apparent velocities recorded below the seabed are an imaging artifact (described in Methods).

As with the 500 kHz multibeams, for flows B and C, the acoustic scattering was clearly masked by heavy attenuation. In addition, for the second pulse in flow E the seabed below was obscured through attenuation at 1,200 kHz that did not show up at 500 kHz.

The attenuating layer limited the closest point to the seabed at which a valid velocity could be obtained within the flow. The height off the seabed at which the attenuation blocks the flow was greater for the ADCP than the multibeam data. This reflects the higher attenuation coefficient at 1,200 kHz, rather than 500 kHz of the multibeams.

### Mechanical profiling

About 10 m behind the instrument package, and just in front of the ADCP, a mechanically winched set of probes on a frame was lowered up and down the lower 15 m of the water column to within 0.5 m of the seabed about every 60 s. The sensors recorded optical backscatter (a proxy for suspended sediment), as well as temperature and salinity.

**Figure 10:**
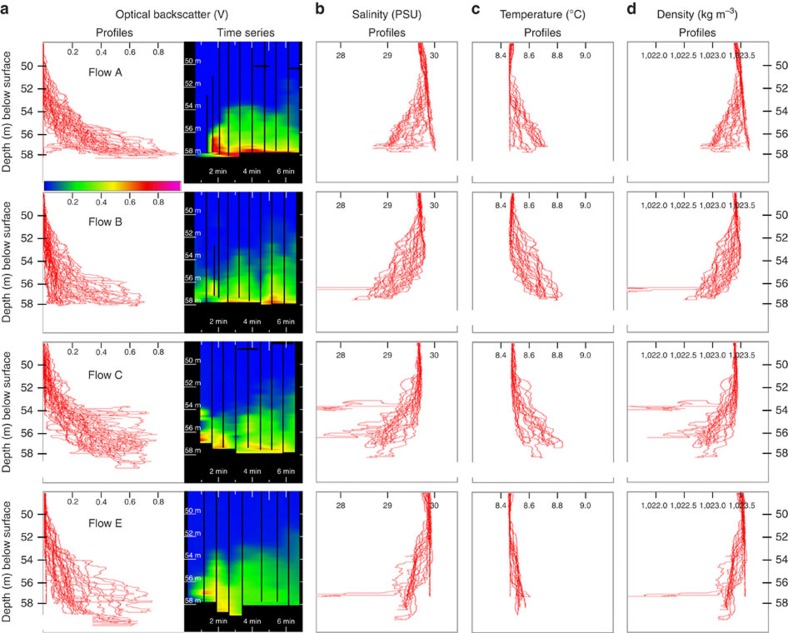
Optical backscatter and hydrography. (**a**) Profiles and time series of optical backscatter strength for a 7-min time window during the strongest four flows (A, B, C and E). Corresponding profiles of (**b**) salinity, (**c**) temperature and (**d**) fluid density (just due to the hydrography) for the same period. Seven-minute time window is identical to that in Fig. 7.

The full 2.3-h time series of optical backscatter is shown in Fig. 6b and indicates that the highest suspended sediment loads (peaking at 0.7 kgm^−3^) were seen during flows A, B, C and E. The suspended sediment structure of the four flows is remarkably similar (Fig. 10), with actually slightly lower levels in the two fastest flows.

For the fastest flow C, the two profiles that occurred during the passing of the attenuating head of the flow did not penetrate to the same depth as other profiles. This was because, although the same length of cable was laid out, the pressure sensor in the package indicates that the sensor did not drop to the same depth. This suggests that the sensor frame was swept downstream at that point. Thus frustratingly, the two opportunities to measure higher suspended sediment loads in the most attenuating part of the flow were not achieved.

### Temperature and salinity profiling

Before the arrival of the first flow of the day, the ambient water at the depth of the sensors had a salinity of 29.8 practical salinity units (PSU) and temperature of 8.45 °C. The completely fresh river water (0 PSU) at the surface had a temperature of 9.95 °C. There is a peak in temperature in the underlying salt water at a depth of 4.0 m of 13.4 °C and 22.7 PSU. Thus, any flow starting at the delta lip at low tide passes through these three water masses in turn. Depending on the level of entrainment, there should be a thermal and salinity anomaly associated with the descending flow.

The temperature and salinity sensors on the probe provides an opportunity to examine the anomalous hydrographic signature of the flow. Every flow exhibited a drop in salinity of up to 1.5 PSU and an increase in temperature of up to 0.3 °C with the anomaly consistently increasing, as the probe descended deeper into the flows (Fig. 10).

Spike-like abrupt drops in the salinity are seen that have no corresponding thermal signature and are believed to be related to bubbles affecting the conductivity probe. As is apparent in the vertically oriented multibeam imagery, the passage of the flow head resulted in abrupt release of ascending bubble clouds.

## Discussion

These data provide an opportunity to unambiguously discern the character of flow that results in CSB migration. The observed surge events only partially modified an existing bedform field with migration of only a fraction of the wavelength. Thus, the characteristic dimensions of the bedforms are predominantly inherited from previous flows. Nevertheless, the bedform character before and after the flow appears the same, suggesting that these flows represent the main mechanism for maintaining these bedforms. By analogy with the bathymetric signature of 103 events in the same area recorded over a 4-month period in 2011 (ref. 28) (Supplementary Movie 1), the dominant mechanism moulding these bedforms typically appears to be upslope displacement of each CSB by just a fraction of a wavelength per low water period.

When attempting to estimate the layer-averaged flow properties, it is necessary to integrate over the full thickness of the flow^6^. A critical unknown is the presence or absence of a higher-density layer at the base of the flow which, if present, will have a disproportionately large influence on the layer-averaged properties. The few previous field measurements of vertical flow structure in comparable high-velocity flows^34^,^35^, used instrumentation that cannot sample close to the base of the flow to distinguish such a layer.

In this study, the density structure of the flow has been inferred through two methods: acoustic scattering from suspended, downward-looking sonars, as well as optical backscatter from a mechanically lower probe. Only the optical probe has been calibrated against physical sampling, but it did not penetrate close enough to the seabed and was deployed insufficiently frequently. The acoustic imaging, however, with a half second update rate, could look qualitatively at the instantaneous suspended sediment distribution. Where the acoustic backscatter signal becomes totally blocked due to attenuation, it suggests an abrupt increase in suspended sediment concentration well in excess of 50 kgm^−3^. This would thus potentially indicate a pycnocline defining an underlying denser basal layer. Such a lower limit of 1.9% by volume, however, does not on its own qualify as a high-density layer as described in the literature (9% would be required for grain-to-grain interaction^36^).

The peak suspended sediment levels observed in flows B and C in the overlying lower-density flow are near-identical to those of the flows without a distinct attenuating basal layer (A, D, E, F and G) and are only high enough in density (∼0.5 kgm^−3^) to explain a flow velocity below ∼0.65 ms^−1^ (assuming common Chezy-type layer-averaged models and without inheriting momentum from upslope). The higher velocities seen in the two fastest flows thus appear to be due to the presence of higher density in a basal layer that presumably is then driving the flow rather than being dragged by the overlying layer.

The absence of such a layer in flow A and E that exhibited comparable velocities might be explained by the fact that the flow was rapidly decelerating (flow A, Supplementary Movie 2) or the more competent part of it was passing by in the adjacent channel (flow E). In both cases, the higher velocity would then reflect the contribution of a high-density layer that either had just decayed (A) or was out of the field of view of the downward-looking sensors (E). The 0.1 m of gradual stoss accretion seen in flow E (Fig. 6a) is assumed to reflect lateral deposition from the more competent flow in the western thalweg.

An additional complication in assessing the excess density of the flow is the density of the entrained ambient fluid relative to the surrounding fluid. The observed drop in salinity and rise in temperature, if not compensated by a suspended sediment load, would make the flow about ∼0.7 kgm^−3^ less dense (Fig. 10d). As the flows at this point in space were not rising, this places a minimum suspended load requirement at the limit of the probe sampling. Thus, the observed density structure that was penetrated by the probe does not appear to have any net density excess. To maintain such speeds therefore, further suggests that the unsampled lower layer must have exhibited significantly greater excess density. Unfortunately, the sampling methodology implemented herein cannot test that hypothesis.

As the attenuating layer boundary fluctuates over a period of a few seconds both vertically and horizontally (Fig. 8; Supplementary Fig. 2), this is taken as further evidence that the horizon is reflecting a gradient within a fluid layer and not a static boundary. The abrupt thickening of the attenuating layer corresponds to the observed location of HJs seen in laboratory experiments^14^. Furthermore, the fact that this thickening starts off well downstream of the base of the lee slope, but moves towards it and reduces in amplitude as the flow wanes, matches laboratory experiments^37^ showing the transition from a flushed to a submerged HJ as the kinematic potential wanes. The abrupt layer thickening is therefore postulated as field evidence of a HJ in a denser basal layer, previously only seen in laboratory experiments^14^. The observed upslope migration implies supercritical bedforms, such as antidunes or cyclic steps. The identification of a HJ better supports the cyclic step origin.

Independent measurements of the peak suspended sediment load in the river water around low tide^30^, clearly indicate that hyperpycnal conditions^38^ do not exist within the plume sufficient to overcome the hydrographic density contrast and thus to drive the fresh water down into the salt. Nevertheless, in the previous year's campaign^31^, rapid profiling and acoustic imaging clearly indicate that the suspended load of the fresh water plume is able to settle out from the fresh water into the salt water thereby avoiding the need to overcome the full density contrast.

The fact that the flows have a faint but distinct brackish and warm anomaly can be used to try and constrain the relative proportion of entrained fluid. If the flow was formed by simple mixing of river water and ambient water, the salinity reduction suggests ∼5% dilution by volume, but the thermal anomaly, however, represents ∼20% by volume. A saltier, warmer mixing origin is thus suggested. A likely source for this is the hotter salty layer just below the main halocline. As the bathymetric change (Fig. 2) indicates that the flow was active from depths of <10 m, it is also likely that the mixing involved contributions from progressively colder, but saltier layers below the thermal maxima as the flow progressed deeper.

In conclusion, previous studies have debated whether dense near-bed layers can exist beneath turbidity currents, and whether the type of crescent-shaped, upslope-migrating bedforms seen at the Squamish delta are diagnostic of cyclic steps in supercritical flow. To address this, this study illustrates a new method involving forward-and downward-looking multibeams that can image these flows far more clearly than previously. Using this approach, this study demonstrates that the active migration of CSBs is uniquely associated with the subset of the turbidity current flows that contain distinct acoustically attenuating near-bed layers. Using indirect arguments based on the amount of acoustic attenuation, together with lack of sufficient density in the overlying layer to generate the observed flow speeds, that layer is inferred to be higher density.

Those flows with an inferred higher-density basal layer appear unique in that the layer interacts with seabed undulations to trigger periodic HJs locked in phase to the base of the lee slope. Only when this occurs does resolvable bedform migration take place. The unambiguous upslope migration and the evidence for a HJ best supports the cyclic step origin for these CSBs.

## Methods

### Suspended instrument geometry

The instruments were suspended below a vessel that was moored using four anchors located outside the active channel floor (Supplementary Fig. 1). The two M3 multibeams operate at 500 kHz and collect data over a 120° sector using 1° receiver channel spacing. Both were mounted in a frame suspended ∼10 m off the channel floor. The fore-aft profiling system used a 3° transmitter and was facing down with the swath aligned along the channel axis (Fig. 4). As a result it covered a profile ∼35 m long (slightly longer than a single bedform). The plan view utilized a forward-looking configuration with a broad transmitter covering a 30° sector in elevation with the receivers covering the 120° sector in azimuth looking out to a 150 m range up and across the channel (Fig. 2). The downward-looking sonar achieved a 2-cm range resolution, whereas the forward-looking sonar used a 10-cm range resolution. An update rate of 0.5 s was used. Both sonars use 1.8° wide receiver beams spaced at ∼1.0°.

About 15 m downstream of the multibeams, a 1,200 kHz ADCP was independently suspended 10 m off the main stoss surface, measuring currents and acoustic volume scattering in 25 cm bins using a 10 ping ensemble every 4 s. Even though the M3 and ADCP were not synchronized, as they differ in centre frequency (500 versus 1,200 kHz), no interference was observed in either of the acoustic data sets.

Between the ADCP and the suspended multibeams, a mechanically winched probe measuring salinity, temperature, pressure and optical backscatter was repetitively cycled at ∼60-s intervals covering from 15 m above the seabed to within∼50 cm of the sediment–water interface (the height of the sensors from the bottom of the frame).

The spacing between the M3 multibeams, the lowered probe and the ADCP was sufficient that there was no contamination of either acoustic imagery due to the presence of the probe. This was confirmed by examining the ADCP and M3 backscatter at the time of lowering, and no acoustic targets were seen.

That same spacing meant that there was a slight timing lag between when the flows arrived under the M3, when they were probed and when the ADCP current measurements were made. In addition, the spacing meant that the ADCP measurement are made over the lee face of the downstream bedform, whereas the probe is on the trailing edge of the stoss face and the M3 examines the whole stoss side, as well as the upstream lee face.

### Optical backscatter calibration

The optical backscatter voltage level has been empirically correlated with suspended sediment samples within the overlying plume. This suggests that the peak voltage measured (1.0 V) corresponds to ∼0.7 kg m^−3^. Nevertheless, the grain size distribution in the plume differs significantly from the grain size recovered from grabs on the channel floor and thus the calibration for those measurements in the turbidity current may be biased and thus the profiles should be regarded strictly, as a relative view of the gradient rather than absolute measures of the density structure. Due to the transient nature of the flow, no physical samples of the actual suspended load were obtained.

### Limitations of mechanically winched sensors

As the CTD and optical backscatter sensors were mounted on the top-end of the frame (for safety and to prevent them being forced into the seafloor sediment), the sensors were physically unable to approach the seafloor closer than 50 cm. Furthermore, once the exact location of the seafloor was established, the sensor was subsequently not lowered to the point of seabed contact to prevent overspooling of the winch drum. An additional problem with the probe is that, for the most competent flow C, it is clear that, although the same amount of cable was paid out, the sensor did not achieve the usual maximum depth strongly suggesting that it was being swept downstream by the drag of the flow.

### Stabilizing the pitching motion of the M3 imagery

The original M3 vertically oriented imagery was distorted by pitching motion of the instrument. The internal motion sensor had a high-frequency noise overprinting the true pitch and thus the rapid pitching motion could not be corrected using these observations. To overcome this, an image correlation technique was developed that minimized the rotational difference between successive frames based on peak correlation of a series of progressively pitch-rotated scenes. In this manner, the change in pitch of the instrument between successive scenes was estimated and used to predict the high-frequency motion. The longer period pitch orientation was preserved by adding back the low-pass filtered version of the pitch.

### Recognizing sidelobe echoes in the vertical M3 imagery

To avoid misinterpretation of the vertical profile multibeam image, artefacts due to the imaging geometry that overprint the actual seabed phenomena are herein explained. Any multibeam receiver beam has sidelobes that lie on either side of the main beam axis. Thus, in the presence of a particularly high-scattering target at one elevation angle, all beams will pick up a secondary echo at that slant range even if off that particular beam boresight. The most notable scattering target is the specular echo directly below the instrument due to the normal incidence echo on the sediment–water interface. As a result, a ring in the image is seen at the minimum slant range to the seafloor. Beyond that ring, the volume scattering from the water mass is potentially corrupted by sidelobe responses from the seafloor at the same slant range at lower elevation angles. Thus, the appearance of the series of sub-parallel lines above and below the seafloor out on the side of the image (for example, Fig. 8a) should not be confused with real water mass or sub surface scattering. They are merely an artifact due to the spacing and magnitude of the receiver sidelobes.

Notably, when the flow goes by, the strong sediment–water interface echo is obscured and thus the minimum slant range ring disappears. The ring reappears as the flow dies away and the sediment–water interface is no longer masked.

### ADCP limitations in spatially varying flow fields

While the ADCP does provide estimates of the current velocities, it is derived from four beams all oriented at mutually orthogonal directions spaced at 20° away from vertical incidence. At a suspended height of 12 m this corresponds to a spacing of over 8 m between the inversely directed beams. As the length scale of the billows seen in the upper part of the flow is shorter than this, the common assumption that watermasses in the beams at the same elevation share the same velocity is not maintained. Thus, the details of the turbulent flow at scales shorter than the beam spacing cannot be resolved. The most extreme case for this is the head of the flow when it first appears in the upstream facing beam. The Doppler shift in that beam does not match that resulting from near stationary watermasses of the other beams. In this case, the ADCP velocity solution is invalid and a gap in the velocity estimates appears. These are portrayed in Fig. 9 as hatched areas. With a head velocity of ∼2–3 ms^−1^, it takes ∼4 s for this gap to pass, which is the duration of the ensemble averaging used.

### ADCP artifacts due to strong relief between the four beams

The raw beam echo intensity time series from the four beams (Supplementary Fig. 3) clearly indicate that the seabed depth varied up to 3.5 m between beams. It is thus clear that the ADCP was suspended above the lee slope of the downstream bedform. As a result, the furthest range interval for which all four beams are examining water and not seabed echoes is limited by the shortest beam distance to the seafloor. That deepest depth, while close to the seabed on the upstream stoss side, corresponds to a watermass that is over 3 m above the seabed on the downstream stoss side. This further reduces the validity of the velocity estimate.

For the case of flow C, the location of the lee slope moved upstream by ∼5 m during the flow. This can be clearly seen in the changing depth to the seafloor in the four individual beam echo traces (Supplementary Fig. 3).

An additional complication with this non-flat seafloor geometry is that there is the potential to report misleading velocities for depth horizons that are beyond the highest beam bottom-tracking depth.

### Misleading ADCP velocity measurements appearing below the seabed

An ADCP provides velocity estimates based on observations at discrete time intervals common to all four beams. The maximum time/range that the observations are taken is user selectable. Unless a bottom track option is selected, there is no assumption about when the echo actually reaches the seafloor. In the configuration used in this experiment, bottom tracking was deliberately not enabled as this assumes a zero velocity seafloor that is not valid if the seabed itself is moving.

In the absence of knowledge about the location of the seabed, echoes at all time intervals until the user-set maximum range (15 m in this case) are interrogated for a valid velocity estimate irrespective of whether the seabed echo has already arrived (Supplementary Fig. 4). The depth below the instrument that a velocity measurement is reported is based on the assumption that the echoes at that time correspond to discrete volumes at that radial distance along the mainlobe axis of each beam. For the case of a time interval after the seabed has passed, that the assumption would appear to place spurious velocity measurements below the seafloor. Normally, after the seabed return, however, the only echoes are outboard sidelobe returns from the stationary seafloor and thus no velocity is recorded.

During the period, however, when the basal layer is moving, those outboard sidelobe echoes will pick up a reduced-amplitude Doppler-shifted echo corresponding to the movement of the top of the basal layer (Supplementary Fig. 4). The amount of the intensity reduction will reflect the two-way sidelobe suppression (∼−40 dB). However, as the basal layer appears to exhibit a scattering strength approaching that of a seafloor, it is thus typically ∼30+ dB higher than suspended sediments. As a result, under the particular geometry of a fast moving highly scattering near-seabed layer (Supplementary Fig. 4), the ADCP generates additional velocity solutions that, when plotted assuming mainlobe geometry, appear to extend both below the attenuating layer and in fact below the seabed.

The strong difference in elevation between the beams further complicates this geometry. There may in fact be valid Doppler shifts after the minimum slant range in the beams pointing towards deeper areas. The same time window, however, for those beams oriented towards shallower locations will contain false velocity information.

In trying to mask out these spurious echoes, the percent good (a manufacturer-applied quality factor), and the error velocity statistics were investigated. None of these qualifiers, however, indicated that these false sub-seabed echoes are less valid than the true above seabed values. The data are deliberately presented and explained here so that they are not misinterpreted to be valid velocity measurements below the top of the attenuating basal layer. This will be a significant complication for future work, investigating comparable near-seabed currents where the flow thickness of interest is smaller than the bedform relief.

### Inferring a lower limit for concentration in the attenuating layer

At levels above ∼5 kgm^−3^, several authors^39^,^40^have identified the effect that attenuation of sound has on the scattering signal from suspended sediments. Indeed, to invert the concentration from the scattering strength, cumulative calculations of the attenuation loss needs to be made for all layers encountered before that of interest. Given that the concentration of the preceding layers is unknown, this represents a challenging problem. Concentrations that have a significant effect, however, are usually only encountered in bottom boundary layers. For the case of this study, unusually high levels are suspected, as it is clear that the strong and distinct echo from the seabed below is completely masked. Where the attenuating layer is well established, the echo from the overlying moving layers appears to truncate over a path length of <1 m.

Two example experimental studies were referenced in this work to try and estimate the likely minimum concentration exceeded, at which the scattered signal starts to drop rapidly with range despite high or even increasing concentration.

Thorne *et al*.^39^, did experiments at 3 MHz using glass spheres (their Fig. 5b), as well as natural particles (their Fig. 6b). They looked at the backscattered pressure from a uniformly mixed volume at a variety of ranges. The path lengths considered ranged from 0.25 to 1.05 m, closely corresponding to the distance over which the attenuation masking was apparent in the field results reported here. Their observations did not go above 3 kgm^−3^, but their matching model predicted two orders of magnitude drop in root mean squared pressure over a distance of 1 m for 10 kgm^−3^.

Shen and Lemmin^40^ utilized a lower frequency of 1 MHz and examined both the backscattered response, as well as the transmitted signal. They used a fixed path length of 0.52 m and investigated concentrations of 29 and 47 kgm^−3^. Notably, they did not achieve complete masking of the signal over that range. As the observation reported here are at the same (1.2 MHz for the ADCP) or lower (0.5 MHz for the M3) frequency, that 47 kgm^−3^ level is taken as a conservative minimum lower limit for the concentration around the top of the attenuating layer. Assuming a grain density of 2,650 kgm^−3^, this corresponds to a volume concentration of ∼1.8%. One way to improve on this estimate would be to undertake tank tests using the instruments with the grain size distribution actually present in the flow.

## Additional information

**How to cite this article:** Hughes Clarke, J. E. First wide-angle view of channelized turbidity currents links migrating cyclic steps to flow characteristics. *Nat. Commun.* 7:11896 doi: 10.1038/ncomms11896 (2016).

## Supplementary Material

Supplementary Information Supplementary Figures 1-4

Supplementary Movie 1 Bathymetric evolution of the Squamish prodelta over summer 2011. 83 frame animation showing 1 to 3 day sequential multibeam surveys of a 600x 400 m area off the front of the Squamish delta. Location indicated in Figure 1. The data were collected in the summer of 2011 (from April 27^th^ (JD117) to August 24^th^ (JD236)). The bathymetry is presented as a 2m resolution sun-illuminated image (from 315T) to highlight the evolving short wavelength relief. The depths range from 2m (just below low water) to 67m. The location and orientation of the 150m radius window of the planiform multibeam (described in the paper, installed in 2013) is superimposed as a yellow arc. The animation illustrates the day to day episodic migration of the crescent shaped bedforms. This indicates that the discrete event seen in 2013 (reported in detail in this paper) which involved a fractional shift of the bedform in one lower low water period, is characteristic of the activity that forms and maintains the CSBs within the channels of the prodelta. The apparent noise in the data is largely sonar mistracking on the gas plumes that were strongly but ephemerally developed on the prodelta slope

Supplementary Movie 2 Plan view of four strongest flows in June 21^st^ 2013. Each frame of this animation, shows a composite of 4 images from flows A, B, C and E on June 21^st^. Each view extends out 150m from the forward looking multibeam showing a plan view of the seabed upstream of the instrument package. For location, see Fig 2 of the main submitted paper, and to understand the imaging geometry, see supplemental figure 1.Note that the imagery consists of individual scenes derived from stacking 2 half second frames to generate one clearer 1 second scene. The data rate is played back at approximately 5x actual speed (exact speed depends on the browser's implementation of the animated gif frame rate). The average propagation rate of the head of the flow C is about 2.5ms^-1^.

Supplementary Movie 3 Downward looking M3 imagery of Flow B. Animation of imagery generated from the downward looking, along-channel oriented multibeam during flow event B. The image consists of individual frames generated at half second intervals. Representative stills of the animation are provided in supplementary figure 2. The animation illustrates the temporal development and instability of the location of the hydraulic jump on the top of the attenuating layer. The jump is located just downstream of the base of the lee slope of the cyclic step bedform.

Supplementary Movie 4 Downward looking imagery of Flow C. Animation of imagery generated from the downward looking, along-channel oriented multibeam during flow event C. The image consists of individual frames generated at half second intervals. Representative stills of the animation are provided in Fig 8 of the submitted paper. The animation illustrates the initial thicker and undulating appearance of the attenuating layer, followed by the development of a more planar interface. It shows the growth and instability of the location of the hydraulic jump developed on that interface, just downstream of the base of the lee slope of the cyclic step bedform.
