# Supplementary material for: First wide-angle view of channelized turbidity currents links migrating cyclic steps to flow characteristics
Source: Nat Commun. 2016 Jun 10;7:11896. doi: 10.1038/ncomms11896 (PMC5438126; doi:10.1038/ncomms11896)
Supplement: Supplementary Information — Supplementary Figures 1-4 [file ncomms11896-s1.pdf]

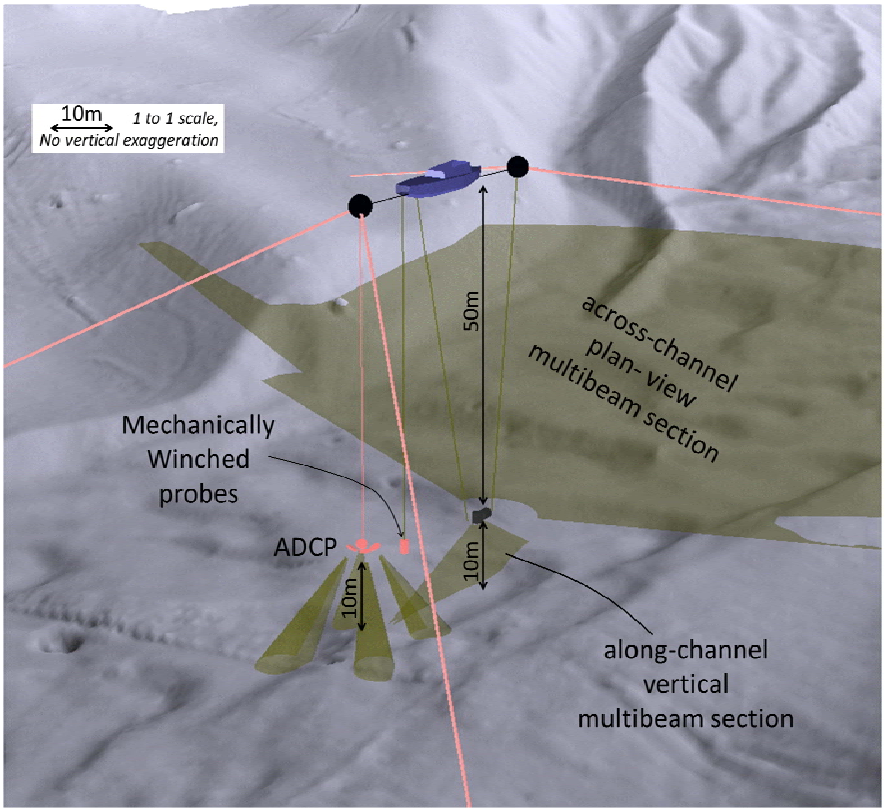

Supplementary Figure 1:

TITLE: Experimental Configuration

Diagram illustrating the layout of the experiment. The vessel was positioned between two buoys which in turn were constrained by two anchors each. This ensured the azimuthal alignment of the instrument package below. The vessel was centred over a submarine channel in 60m of water facing upstream. The two M3 multibeams were mounted on a frame suspended 10m off the seabed and aligned along the channel axis. One was oriented forward looking in plan view, and the other downward looking in along channel section. Off the stern of the vessel, a mechanically winched instrument package was lowered into the flow at 60 second intervals, normally reaching to within ~ 50cm of the seabed. A 1200 kHz ADCP was suspended below the downstream buoy and located 10m off the seabed.

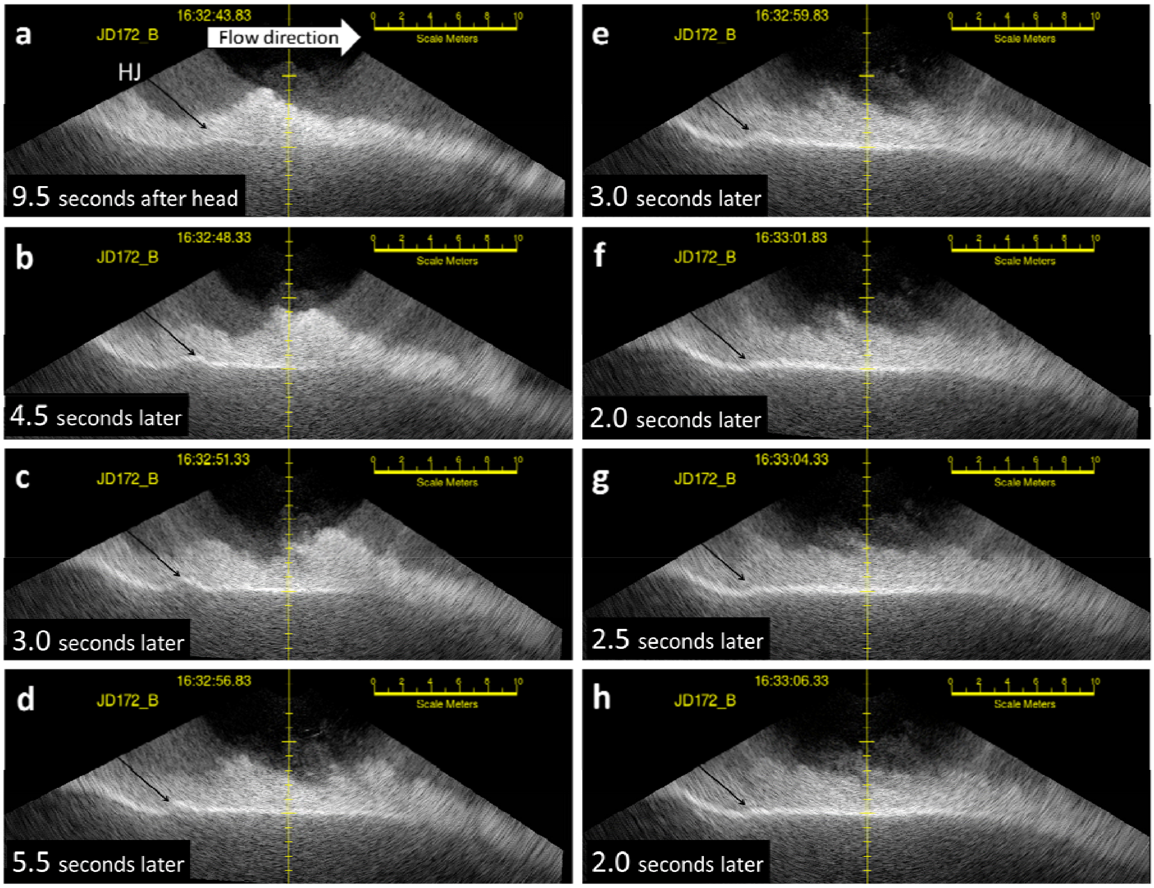

Supplementary Figure 2:

TITLE: Development of Hydraulic Jump in Flow B

For flow B, eight still frame images illustrate the along channel section over the length of a single bedform. Flow from left to right. The successive frames all fall within a 23 second window when the hydraulic jump at the top of the acoustically-attenuating basal layer is best developed. As time progresses, the amplitude of the hydraulic jump (indicated by black arrow) wanes and migrates towards the base of the lee slope. Unlike flow C, for this flow, no resolvable net migration of the lee slope was noted.

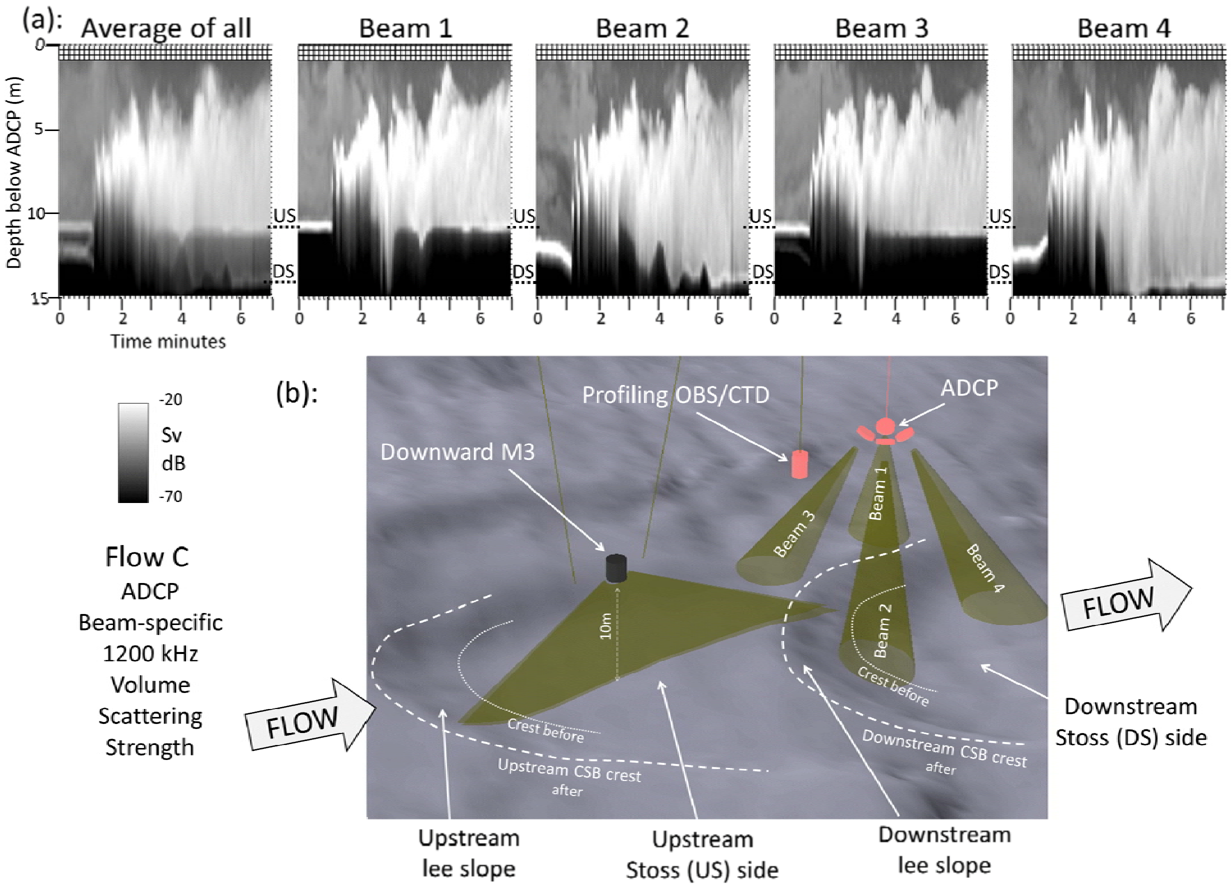

Supplementary Figure 3:

<u>TITLE: Variation in seabed echo range for the four ADCP beams.</u>

a: time series plots showing 15m equivalent vertical range time series of ADCP intensities for each of the 4 beams as well as the average of the four. US: Upstream Stoss, DS: Downstream Stoss.

b: 3D graphic illustrating the geometry and placement of the four mutually orthogonal ADCP beams compared to the crescentic bedform crests (as they were positioned both before and after Flow C) and the M3 downward looking profile.

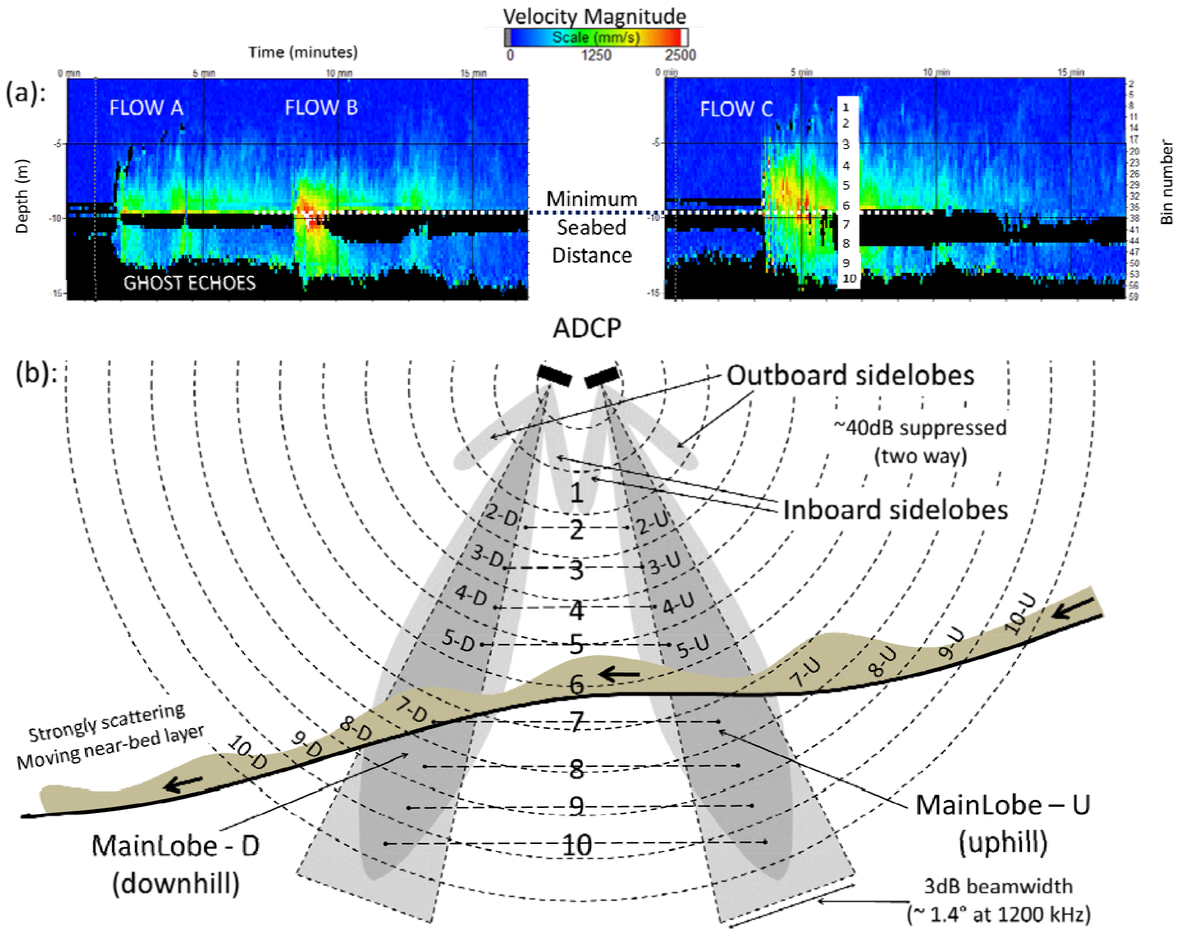

Supplementary Figure 4:

<u>TITLE: Why velocity structure below the seabed is reported.</u>

a: Full 15m profiles of reported velocities for Flows A, B and C. Minimum seabed distance (on upstream stoss side) is indicated. The ghost velocity structure appears below the seabed just when the near seabed velocities are active.

b: Illustrative diagram showing the actual and assumed geometric relationship for each of the ADCP velocity layers. In this case layers 1 to 5 are valid. Layer 6 is not reported due to inboard sidelobe contamination by normal incidence seabed echoes. Layers 7 to 10 are reported below the seabed, even though they actual represent motion from outboard sidelobes picking up the actively moving near-seabed layer. For clarity, just two opposing azimuth beams are illustrated indicated by the suffix U (upslope) and D (downslope).
