# Supplementary figures and images for: First wide-angle view of channelized turbidity currents links migrating cyclic steps to flow characteristics
Source: Nat Commun. 2016 Jun 10;7:11896. doi: 10.1038/ncomms11896 (PMC5438126; doi:10.1038/ncomms11896)

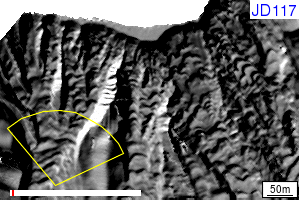

Supplement: Supplementary Movie 1 — Bathymetric evolution of the Squamish prodelta over summer 2011. 83 frame animation showing 1 to 3 day sequential multibeam surveys of a 600x 400 m area off the front of the Squamish delta. Location indicated in Figure 1. The data were collected in the summer of 2011 (from April 27th (JD117) to August 24th (JD236)). The bathymetry is presented as a 2m resolution sun-illuminated image (from 315T) to highlight the evolving short wavelength relief. The depths range from 2m (just below low water) to 67m. The location and orientation of the 150m radius window of the planiform multibeam (described in the paper, installed in 2013) is superimposed as a yellow arc. The animation illustrates the day to day episodic migration of the crescent shaped bedforms. This indicates that the discrete event seen in 2013 (reported in detail in this paper) which involved a fractional shift of the bedform in one lower low water period, is characteristic of the activity that forms and maintains the CSBs within the channels of the prodelta. The apparent noise in the data is largely sonar mistracking on the gas plumes that were strongly but ephemerally developed on the prodelta slope [file ncomms11896-s2.gif]

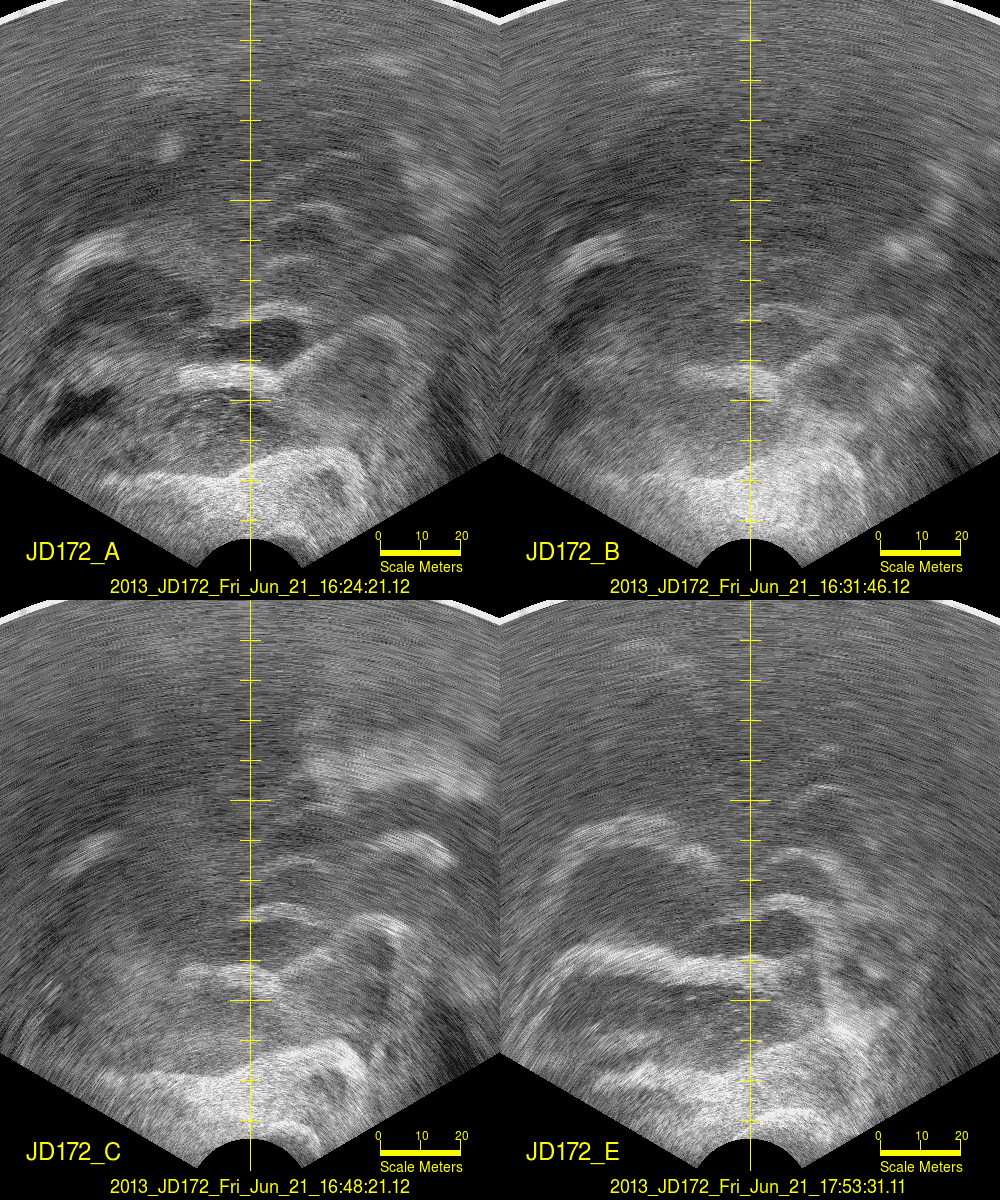

Supplement: Supplementary Movie 2 — Plan view of four strongest flows in June 21st 2013. Each frame of this animation, shows a composite of 4 images from flows A, B, C and E on June 21st. Each view extends out 150m from the forward looking multibeam showing a plan view of the seabed upstream of the instrument package. For location, see Fig 2 of the main submitted paper, and to understand the imaging geometry, see supplemental figure 1.Note that the imagery consists of individual scenes derived from stacking 2 half second frames to generate one clearer 1 second scene. The data rate is played back at approximately 5x actual speed (exact speed depends on the browser's implementation of the animated gif frame rate). The average propagation rate of the head of the flow C is about 2.5ms-1. [file ncomms11896-s3.gif]

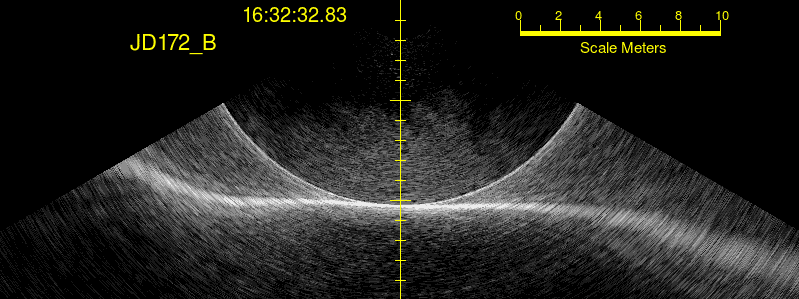

Supplement: Supplementary Movie 3 — Downward looking M3 imagery of Flow B. Animation of imagery generated from the downward looking, along-channel oriented multibeam during flow event B. The image consists of individual frames generated at half second intervals. Representative stills of the animation are provided in supplementary figure 2. The animation illustrates the temporal development and instability of the location of the hydraulic jump on the top of the attenuating layer. The jump is located just downstream of the base of the lee slope of the cyclic step bedform. [file ncomms11896-s4.gif]

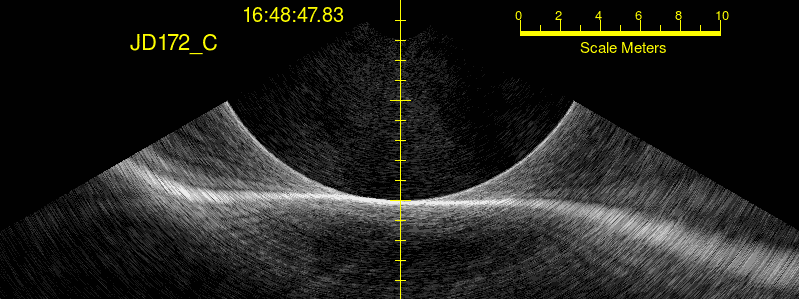

Supplement: Supplementary Movie 4 — Downward looking imagery of Flow C. Animation of imagery generated from the downward looking, along-channel oriented multibeam during flow event C. The image consists of individual frames generated at half second intervals. Representative stills of the animation are provided in Fig 8 of the submitted paper. The animation illustrates the initial thicker and undulating appearance of the attenuating layer, followed by the development of a more planar interface. It shows the growth and instability of the location of the hydraulic jump developed on that interface, just downstream of the base of the lee slope of the cyclic step bedform. [file ncomms11896-s5.gif]
